# Dynamic changes of serum α-fetoprotein predict the prognosis of bevacizumab plus immunotherapy in hepatocellular carcinoma

**DOI:** 10.1097/JS9.0000000000001860

**Published:** 2024-06-21

**Authors:** Zhenyun Yang, Yizhen Fu, Qianyu Wang, Yangxun Pan, Juncheng Wang, Jinbin Chen, Dandan Hu, Zhongguo Zhou, Minshan Chen, Yaojun Zhang

**Affiliations:** a Department of Liver Surgery, Sun Yat-sen University Cancer Center; b Collaborative Innovation Center for Cancer Medicine, State Key Laboratory of Oncology in South China, Sun Yat-Sen University Cancer Center; c Guangdong Provincial Clinical Research Center for Cancer, Sun Yat-sen University Cancer Center, Guangzhou, Guangdong; d Key Laboratory of Carcinogenesis and Translational Research (Ministry of Education/Beijing), Laboratory of Molecular Oncology, Peking University Cancer Hospital and Institute, Beijing, People’s Republic of China

**Keywords:** AFP trajectory, bevacizumab, hepatocellular carcinoma, immunotherapy, survival

## Abstract

**Background:**

Alpha-fetoprotein (AFP) has been established as a biomarker for hepatocellular carcinoma (HCC); however, whether its dynamic changes could predict the response to systemic therapy remains elusive. This study explored the AFP trajectory and the association with survival in patients who received bevacizumab plus immunotherapy.

**Materials and Methods:**

We retrospectively enrolled 536 HCC patients who received bevacizumab plus immunotherapy between February 2021 and February 2023. Patients were divided into two groups according to AFP values before treatment (400 ng/ml). Dynamic changes of AFP were fitted using a latent class model to generate the AFP trajectories. Multivariable Cox models were utilized to compute hazard ratios (HRs) for survival. Inverse-probability-of-treatment weighted analyses were conducted to mitigate the influence of unmeasured confounding variables. The primary endpoint is progression-free survival (PFS). The second endpoint is overall survival (OS).

**Results:**

Three distinct trajectories were identified for AFP-low and AFP-high patients, respectively. In the AFP-low group, compared with the high-rising class (25%; *n*=69), HRs of PFS were 0.39 and 0.2 for the low-stable class (59.1%; *n*=163) and sharp-falling class (15.9%; *n*=44), after adjusting by tumor diameter, tumor number, and extra-hepatic metastasis. In the AFP-high group, compared with the high-stable class (18.5%; *n*=48), HRs of PFS were 0.3 and 0.04 for the middle-stable class (56.5%; *n*=147) and sharp-falling class (25%; *n*=65), after adjusting by tumor diameter, tumor number, and extra-hepatic metastasis. Furthermore, the AFP trajectories exhibited the utmost relative importance among all covariates regarding PFS and OS in the multivariable regression models.

**Conclusion:**

The AFP trajectories in HCC patients receiving bevacizumab and immunotherapy constituted an independent biomarker indicative of clinical outcomes. Findings from this study hold potential clinical utility in dynamically forecasting the prognosis of systemic therapy in HCC patients and facilitating clinical decision-making. Rapid reduction of AFP post-treatment can lead to favorable patient prognoses.

## Introduction

Highlights

- This study incorporated an expanded real-world study cohort in China, comprising a total of 536 patients (bevacizumab plus immunotherapy).
- Directing attention toward alpha-fetoprotein (AFP) dynamic changes as opposed to its singular value and the delineation of AFP trajectories encompassing both AFP-low and AFP-high patient subgroups.
- The AFP trajectories in hepatocellular carcinoma (HCC) patients receiving bevacizumab and immunotherapy constituted an independent biomarker indicative of clinical outcomes.
- Findings from this study hold potential clinical utility in dynamically forecasting the efficacy of immunotherapy combined with antiangiogenic therapy in HCC patients.

Hepatocellular carcinoma (HCC) emerges as a preeminent malignant neoplasm on a global scale and holds a position among the top three leading causes of cancer-related mortality^1^. In light of current circumstances, prognostic models suggest an excess of one million novel instances of HCC, and a commensurate number of HCC-associated deaths are envisaged by the year 2040^2^. Unfortunately, due to the clandestine onset of HCC, a significant proportion of 70–80% of patients are met with their initial diagnosis at an intermediate or advanced disease stage, thereby circumscribing their therapeutic choices to nonoperative interventions, accentuating the pressing demand for efficacious systemic therapies^3–5^.

In various cancer contexts, immunotherapy has emerged as a promising therapeutic modality. The combination of immunotherapy and antiangiogenic therapy has been proven to be rather effective in advanced-stage HCC patients. For example, anti-programmed death ligand 1 (anti-PD-L1) agent atezolizumab plus anti-vascular endothelial growth factor (anti-VEGF) agent bevacizumab and anti-programmed death 1 (anti-PD1) agent sintilimab plus bevacizumab both have shown significant enhancements in overall survival (OS), progression-free survival (PFS), objective response rate (ORR), and quality of life when compared to sorafenib^6,7^. However, there is still a lack of sensitive indicators for early prediction of treatment efficacy.

Alpha-fetoprotein (AFP), the inaugural oncofetal biomarker delineated in patients with HCC, stands as the predominant tool utilized for the detection and longitudinal monitoring of individuals afflicted with HCC. Elevated AFP levels exhibit a correlative association with an unfavorable prognosis across diverse clinical settings^8–12^. This biomarker assumes pivotal significance in prognosticating tumor recurrence post-hepatic resection and in identifying optimal candidates for hepatic transplantation^8,9^. Meanwhile, baseline AFP concentrations have demonstrated efficacy in prognosticating survival outcomes in locoregional and systemic therapeutic modalities^10–12^. Furthermore, the transient AFP response is employed for the prognostication of radiologic response and survival in patients with HCC undergoing systemic chemotherapy^13–18^. Nevertheless, the role of AFP fluctuation, which encompasses change rate and time interval, remains ambiguous and inadequately defined, necessitating a pressing need to elucidate the potential trajectories of AFP in advanced HCC. Within the context of this longitudinal, retrospective, real-world study, our objective is to delineate the dynamic changes of AFP using trajectory models and evaluate its influence on clinical outcomes in HCC patients treated with bevacizumab plus immunotherapy, and clinicians can rely on the observation of AFP fluctuations in lieu of complex calculations, thereby facilitating clinical decision-making.

## Materials and methods

### Patients and data collection

**Figure 1:**
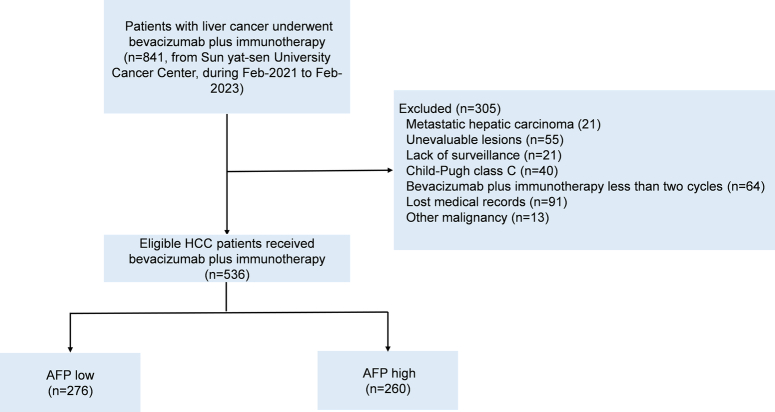
The flowchart of patients. AFP, alpha-fetoprotein; HCC, hepatocellular carcinoma.

Between February 2021 and February 2023, 841 patients diagnosed with advanced HCC who received bevacizumab plus atezolizumab or sintilimab were selected from Sun Yat-sen University Cancer Center. The exclusion criteria were as follows: (1) Metastatic hepatic carcinoma; (2) Unevaluable lesions; (3) Lack of surveillance; (4) Child–Pugh class C; (5) Lost medical records; and (6) Other malignancy. Furthermore, in order to dynamically observe the changes in serum AFP, only patients with pre-treatment and at least two post-treatment AFP records would be enrolled in this study. Finally, 536 HCC patients were enrolled in the study (Fig. 1). The retrospective analysis received approval from the Clinical Research Ethics Committee of our cancer center (Approval Number: B2023-673-01). The study was retrospectively registered at ResearchRegistry.com, and this cohort study complied with the STROCCS statement^19^.

### AFP measurement

All AFP measurements are performed by professionals in the Department of Medicine Laboratory of our hospital in accordance with standardized operating procedures, and a formal test report is issued. The upper limit of the normal value of serum AFP is 25 ng/ml, and the upper limit of the instrument measurement value is 121 000 ng/ml; hence values beyond the measurement range are uniformly recorded and analyzed as 121 000 ng/ml. Patients would receive routine AFP measurement within 1 week before the initiation of bevacizumab plus immunotherapy, and every 2 months, the re-examination of AFP along with computed tomography (CT) or magnetic resonance imaging (MRI) scans during the treatment period for the evaluation of treatment responses.

### Model construction

Since the numerical distribution of AFP values is skewed (Supplementary Fig. S2, Supplemental Digital Content 2, http://links.lww.com/JS9/C820), log transformations are applied to the values for a normal distribution. The dynamic changes, or the trajectories of AFP value, are fitted using the latent class trajectory modeling algorithm described below.

Firstly, a latent class linear mixed model (LCMM) is conducted, which assumes that the AFP value has Gaussian random deviations (random effects, correlated errors, and measurement errors) and the covariate effects (time point of measurement, disease progression, etc.) are constant across the entire range of AFP values (0–121 000 ng/ml)^20,21^. The LCMM of AFP value is established through the following three steps^20,22^. In step 1, we initially built a scoping simple fixed model and set the classes of trajectories from 2 to 5 based on previous literature^23,24^. The optimal number of classes is based on the lowest Bayesian information criteria (BIC) with more than 10% participants in any single trajectory class. Step 2, refine the model using the favored number of classes determined in step 1, testing for the following model variants: (1) fixed intercept and slope, (2) random intercept, and (3) random intercepts and random slopes. Due to the potential risk for a local maximum rather than a global maximum in mixture modeling, each model would be executed multiple times (100 times) with different random starting values. Among these iterations, the solution with the highest log-likelihood is selected and presented. Step 3, choose the best one from the above three variant types based on the model adequacy assessments, which are determined by log-likelihood (the higher, the better), relative entropy (the closer to one, the better), and average posterior probability of assignments (APPA) (above 70% in all classes). Both the log-likelihood and relative entropy will be calculated and presented during the model construction, and the APPA refers to the average value obtained by calculating the maximum posterior probability of assignment for each participant across all trajectory classes.

However, due to the assumption of Gaussian random deviations and constant covariate effects may not hold for AFP dynamic change, the latent process mixed model (LPMM) is then performed^25,26^. Due to the unknown link between the outcomes (AFP trajectory) and the underlying latent process, an additional parameter *link function* is used to fix the latent process dimension, with *link*=‘linear’ for a linear transformation, *link*=‘beta’ for a rescaled *β* cumulative distribution function and *link*=‘splines’ for an *I*-splines transformation, and the function form with the lowest BIC is favored. Similar to the LCMM, we set the number of trajectories from 2 to 5 and identified the model with the smallest BIC value and at least 5% of participants in any single class as the optimal model.

Finally, by comparing the advantages and disadvantages of the LCMM and LPMM models based on various parameters (BIC, log-likelihood, APPA, and at least 10% participants in any single trajectory class), goodness of curves fitting, consistency with clinical experience and logic in terms of trends, and whether the trajectory models can guide clinical practice, we ultimately decide which model to adopt.

In addition, since the significant differences in the distributions as well as the clear variations of AFP among HCC individuals, and with only about 50–60% of HCC patients undergoing elevated AFP, we are concerned that HCC tumors may exhibit different biological characteristics and sensitivities to bevacizumab plus immunotherapy treatment among individuals with varying AFP levels. Therefore, we have divided patients into two groups, AFP-high and AFP-low, using 400 ng/ml as the threshold, and performed model construction and following analysis separately.

### Outcomes

The primary endpoint is progression-free survival (PFS) after receiving the combination therapy of bevacizumab and immunotherapy. PFS is defined as the interval from the start of treatment to disease progression, death, or the last follow-up. The second endpoint is overall survival (OS), which is defined as the time interval from the start of treatment to cancer-related death or the last follow-up. Response Evaluation Criteria in Solid Tumors (RECIST, version 1.1) was used to assess the tumor response^27^.

### Literature search strategy

We searched PubMed, Google Scholar, and relevant journals using the following keywords: ‘AFP,’ ‘dynamic changes,’ ‘bevacizumab,’ ‘immunotherapy,’ and ‘HCC.’ We included original research articles, reviews, and case reports published in English between 2000 and 2024. Additionally, citation chaining and reference lists were used to find additional pertinent articles. The inclusion and exclusion criteria were defined to ensure that only high-quality and relevant studies were included in the manuscript.

### Statistical analysis

Characteristics across different groups were compared using unpaired Student’s *t* test, Mann–Whitney *U* test, *χ* ^2^ test or Kruskal–Wallis tests for continuous variables. Categorical data underwent analysis via Pearson’s *χ* ^2^ test or Fisher’s exact probability test. Kaplan–Meier curves were constructed to illustrate OS and PFS, and survival analysis was conducted using the log-rank test. The construction of AFP trajectory was conducted using the R package LCMM (version 2.1.0) in R. Cox proportional hazard models were utilized to investigate the correlation between AFP trajectories and clinical outcomes, with adjustments made for tumor diameter, tumor number, and extra-hepatic metastasis. In order to address potential unmeasured confounding factors, an inverse-probability-of-treatment weighted analysis (IPTW) was conducted through marginal structural models. This involved the use of predicted probabilities, calculated based on tumor diameter, tumor number, and extra-hepatic metastasis, to assess the stabilized inverse-probability-of-treatment weight. The relative importance of each parameter to risk of clinical outcomes was evaluated utilizing Harrell’s RMS R package. Statistical significance was defined as *P* values <0.05. All statistical analyses were executed using Statistical Product and Service Solutions (SPSS version 25.0, Inc., Chicago, USA) and R software (R version 4.3.0, R Foundation, Vienna, Austria).

## Results

### Characteristics of patients

Between February 2021 and February 2023, a cohort of 536 patients diagnosed with HCC and treated with bevacizumab plus immunotherapy (atezolizumab or sintilimab) was identified from Sun Yat-sen University Cancer Center. In this study, 290 patients received bevacizumab plus sintilimab (Bev-Sinti), and 246 patients received bevacizumab plus atezolizumab (Bev-Atezo). No significant difference in baseline features was observed between the Bev-Sinti and Bev-Atezo cohorts (Supplementary Table S1, Supplemental Digital Content 4, http://links.lww.com/JS9/C822). Meanwhile, the outcomes were similar in the two groups (Supplementary Fig. S1, Supplemental Digital Content 1, http://links.lww.com/JS9/C819). Figure 1 delineated the enrollment process. The median AFP value was 355.45 ng/ml. The mean AFP measurement time was 5.4 (range 3–34). The median follow-up duration was 12.6 months, during which 124 patients died.

In this study, we have divided patients into two groups, AFP-low (276 cases) and AFP-high (260 cases), using 400 ng/ml as the threshold. Disparities in baseline characteristics were apparent between the AFP-low and AFP-high groups (Supplementary Table S2, Supplemental Digital Content 5, http://links.lww.com/JS9/C823), which verified the necessity of grouping according to AFP level. Furthermore, it was noted that the AFP-low group exhibited extended median PFS and OS compared to the AFP-high group (PFS: *P*=0.039; OS: *P*=0.0045) (Supplementary Fig. S3, Supplemental Digital Content 3, http://links.lww.com/JS9/C821), thereby validating the imperative of stratifying patients based on AFP levels. Differences were observed in baseline characteristics among three AFP trajectory groups rather than in APF-low or AFP-high patients, as shown in Tables 1, 2.

**Table 1:** Baseline characteristics of AFP-low patients

Variables	High-rising (*n*=69)	Low-stable (*n*=163)	Sharp-falling (*n*=44)	*P*	High-rising vs. low-stable	High-rising vs. sharp-falling	Low-stable vs sharp-falling
Age, years	54±10.5	55.1±10	57.6±12.1	0.197	0.074	0.444	0.165
Sex				0.330	0.948	0.220	0.161
Male	62 (89.9)	146 (89.6)	36 (81.8)				
Female	7 (10.1)	17 (10.4)	8 (18.2)				
Hepatitis infection				0.749	0.537	0.496	0.787
Yes	58 (84.1)	142 (87.1)	39 (88.6)				
No	11 (15.9)	21 (12.9)	5 (11.4)				
ALBI grade				0.534	0.399	0.646	0.774
I	47 (68.1)	112 (68.7)	32 (72.7)				
II	20 (29)	50 (30.7)	12 (27.3)				
III	2 (2.9)	1 (0.6)	0 (0)				
Tumor diameter (cm)	7.1±4.5	6.8±4.6	9.1±4.2	0.010	0.021	0.638	0.003
Tumor number				0.001	0.009	<0.0001	0.03
Single	12 (17.4)	56 (34.4)	23 (52.3)				
Multiple	57 (82.6)	107 (65.6)	21 (47.7)				
Macrovascular invasion				0.263	0.783	0.223	0.104
Yes	28 (40.6)	63 (38.7)	23 (52.3)				
No	41 (59.4)	100 (61.3)	21 (47.7)				
Extra‑hepatic metastasis				0.013	0.031	0.005	0.162
Yes	39 (56.5)	67 (41.1)	13 (29.5)				
No	30 (43.5)	96 (58.9)	31 (70.5)				
ALT, IU/l	34.6 (5.8–199.1)	35.9 (11.1–316.6)	37.7 (5.2–316.6)	0.590	0.714	0.313	0.662
AST, IU/l	44 (6.9–249.2)	46.4 (18.8–233.5)	43.4 (16.3–432.7)	0.711	0.600	0.507	0.972
Albumin, g/l	43.5 (27.5–53.4)	42.9 (31.4–51.1)	42.3 (21–54.2)	0.362	0.679	0.520	0.311
TBil, μmol/l	12.3 (5–110)	15.3 (5.9–37.2)	13.8 (3.4–235.1)	0.158	0.900	0.990	0.888
WBC, ×10^9^/l	6.6 (2.2–13.5)	6.6 (2–11.7)	6.7 (3.1–17.1)	0.897	0.937	0.623	0.614
Hemoglobin, g/l	141 (75–170)	141.5 (100–181)	143 (83–181)	0.887	0.457	0.521	0.762
Platelet, ×10^9^/l	162 (58–643)	178.5 (47–446)	186 (61–647)	0.634	0.680	0.719	0.440
PT, s	12 (9.8–18.3)	12 (10.6–14.4)	11.9 (10.1–17.5)	0.660	0.925	0.528	0.670

*Note*: Data are presented as mean±SD, median (range), or *n* (%). AFP-low: AFP <400 ng/ml, *χ* ^2^ test (or Fisher’s exact probability test) was performed for categorical measures, and the Kruskal–Wallis test for continuous measures; Bonferroni method was used to correct *P* values for multiple comparisons; *P*<0.05 is statistically significant.

AFP, alpha‑fetoprotein; ALBI grade, albumin–bilirubin grade; ALT, alanine aminotransferase; AST, aspartate aminotransferase; TBil, total bilirubin; WBC, white blood cell; PT, prothrombin time.

**Table 2:** Baseline characteristics of AFP-high patients

Variables	High-stable (*n*=48)	Middle-stable (*n*=147)	Sharp-falling (*n*=65)	*P*	High-stable vs. middle-stable	High-stable vs. sharp-falling	Middle-stable vs. sharp-falling
Age, years	48.7±11.4	52.2±12.7	53±10.3	0.124	0.073	0.055	0.653
Sex				0.723	0.490	0.912	0.538
Male	41 (85.4)	131 (89.1)	56 (86.2)				
Female	7 (14.6)	16 (10.9)	9 (13.8)				
Hepatitis infection				0.116	0.184	0.071	0.361
Yes	46 (95.8)	129 (87.8)	54 (83.1)				
No	2 (4.2)	18 (12.2)	11 (16.9)				
ALBI grade				0.207	0.197	0.05	0.613
I	26 (54.2)	98 (66.7)	47 (72.3)				
II	22 (45.8)	47 (32)	18 (27.7)				
III	0 (0)	2 (1.4)	0 (0)				
Tumor diameter (cm)	11.3±5.8	8.2±4.5	8.9±4.3	<0.0001	<0.0001	0.008	0.280
Tumor number				0.002	0.867	0.018	0.001
Single	8 (16.7)	23 (15.6)	24 (36.9)				
Multiple	40 (83.3)	124 (84.4)	41 (63.1)				
Macrovascular invasion				0.680	0.914	0.464	0.418
Yes	27 (56.2)	84 (57.1)	41 (63.1)				
No	21 (43.8)	63 (42.9)	24 (36.9)				
Extra‑hepatic metastasis				0.031	0.074	0.008	0.166
Yes	29 (60.4)	67 (45.6)	23 (35.4)				
No	19 (39.6)	80 (54.4)	42 (64.6)				
ALT, IU/l	36 (10.6–155.7)	41.4 (3.3–268.2)	41.5 (13.7–257.4)	0.545	0.401	0.115	0.281
AST, IU/l	77.3 (21.5–330.2)	62.3 (15.5–368.7)	50.3 (15.2–373.5)	0.301	0.14	0.181	0.296
Albumin, g/l	40.8 (29.7–51.7)	41.5 (28.4–51.1)	43 (27.9–52.8)	0.390	0.209	0.100	0.056
TBil, μmol/l	21.2 (5.3–52.4)	14.8 (4.7–349.1)	15.5 (4.7–153.4)	0.604	0.560	0.568	0.936
WBC, ×10^9^/l	6.7 (2.4–15.5)	6.3 (2.5–14.8)	6.9 (2.6–20.4)	0.311	0.430	0.521	0.09
Hemoglobin, g/l	142.5 (100–185)	138 (76–199)	143 (102–201)	0.251	0.085	0.650	0.130
Platelet, ×10^9^/l	235.5 (58–528)	187 (53–643)	216 (52–468)	0.071	0.080	0.352	0.443
PT, s	12.2 (10.3–15.9)	12.1 (9.9–15.5)	11.9 (10.2–18.3)	0.145	0.149	0.253	0.879

*Note*: Data are presented as mean±SD, median (range), or *n* (%). AFP-high: AFP ≥400 ng/ml, *χ* ^2^ test (or Fisher’s exact probability test) was performed for categorical measures, and the Kruskal–Wallis test for continuous measures; Bonferroni method was used to correct *P* values for multiple comparisons; *P*<0.05 is statistically significant.

AFP, alpha‑fetoprotein; ALBI grade, albumin–bilirubin grade; ALT, alanine aminotransferase; AST, aspartate aminotransferase; PT, prothrombin time; TBil, total bilirubin; WBC, white blood cell.

### Construction of AFP trajectories

As shown in Supplementary Table S3 (Supplemental Digital Content 6, http://links.lww.com/JS9/C824) and Supplementary Table S4 (Supplemental Digital Content 7, http://links.lww.com/JS9/C825), we set the classes of trajectories from 2 to 5 and established LCMM and LPMM models with fixed intercept and slope, random intercept, random intercepts and random slopes, linear transformation, *β* cumulative distribution, *I*-splines transformation, respectively. Then, according to relative entropy, log-likelihood, BIC value, and percentage of patients in any single class, we finally confirmed that an LPMM model with rescaled *β* cumulative distribution was ideal for AFP trajectories of APF-low group, and an LPMM model with five knot splines transformation was best fitted for the AFP-high group.

**Figure 2:**
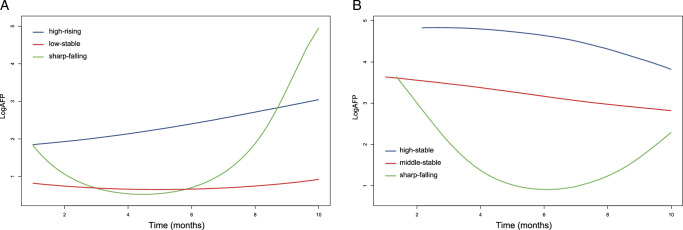
Trajectories of serum AFP in HCC patients after bevacizumab plus immunotherapy. (A) Trajectories of serum AFP in AFP-low group; (B) trajectories of serum AFP in AFP-high group. AFP, alpha-fetoprotein; HCC, hepatocellular carcinoma.

The predicted trajectories of dynamic serum AFP changes of the AFP-low group were delineated in Figure 2A. Three distinct trajectories were identified and classified as follows: high-rising (25%; *n*=69), low-stable (59.1%; *n*=163), and sharp-falling (15.9%; *n*=44). In the high-rising group, AFP exhibited a gradual increase from an initially elevated pre-treatment level toward a higher magnitude. Within the low-stable group, AFP levels remained steady within the range of 0–10 ng/ml before and after initial treatment. In the sharp-falling group, AFP demonstrated a swift decline from a heightened preoperative status (>100 ng/ml) to fall within the 0–10 ng/ml within 4 months of treatment, then exhibited a gradual increase and finally exceeded the other two groups.

In the AFP-high group, the predicted trajectories of dynamic serum AFP changes were shown in Figure 2B. Three discernible trajectories have been delineated and denoted as follows: high-stable (18.5%; *n*=48), middle-stable (56.5%; *n*=147), and sharp-falling (25%; *n*=65). In the high-stable group, AFP levels persist at a heightened state (>10 000 ng/ml). Similarly, the middle-stable group maintains AFP concentrations within the range of 400–10 000 ng/ml. Notably, the sharp-falling trajectory showcases a rapid decline of AFP from an initially elevated preoperative level (>1000 ng/ml) to a range of 0–10 ng/ml within 4 months of treatment and then exhibited a gradual increase, but not surpassing the other two groups.

### Association between AFP trajectories and clinical outcomes

**Figure 3:**
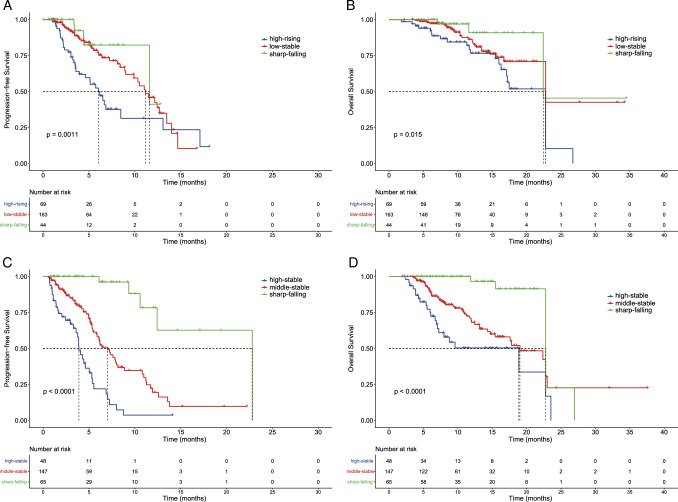
Kaplan–Meier curves of PFS and OS in patients with HCC after bevacizumab plus immunotherapy. (A) PFS in AFP-low group; (B) OS in AFP-low group; (C) PFS in AFP-high group; (D) OS in AFP-high group. AFP, alpha-fetoprotein; HCC, hepatocellular carcinoma; PFS, progression-free survival; OS, overall survival.

Next, we computed the PFS and OS durations for each trajectory subgroup. Within the AFP-low cohort, the median PFS times were determined as 6 months (95% CI: 4.3–7.8), 11.2 months (95% CI: 9.3–3), and 11.6 months (95% CI: 4.6–18.6) for the high-rising, low-stable, and sharp-falling group, respectively (*P*=0.0011). (Fig. 3A). Concurrently, the median OS of the low-stable group exceeded that of the high-rising group but fell short of the sharp-falling group (*P*=0.015), as illustrated in Figure 3B. Table 3 presented the multivariate hazard ratio (HR) of PFS and OS across different AFP trajectory categories. The low-stable group exhibited a reduced risk of disease progression and mortality in comparison to the high-rising group, while the sharpest decline in risk was observed in the sharp-falling group in the unadjusted model. Upon adjusting for tumor diameter, tumor number, and extra-hepatic metastasis, similar results were observed between AFP trajectory groups. Meanwhile, the baseline characteristics after IPTW were shown in Supplementary Table S5 (Supplemental Digital Content 8, http://links.lww.com/JS9/C826).

**Table 3:** AFP trajectory groups and multivariate hazard ratios of clinical outcomes in APF-low patients

	Event/*N*	Unadjusted	Model I
PFS			
High-rising	34/69	1	1
Low-stable	41/163	0.42 (0.31–0.56)	0.39 (0.31–0.49)
Sharp-falling	5/44	0.22 (0.15–0.31)	0.2 (0.13–0.29)
OS			
High-rising	23/69	1	1
Low-stable	25/163	0.77 (0.56–1)	0.8 (0.61–1)
Sharp-falling	3/44	0.22 (0.13–0.34)	0.23 (0.12–0.41)

AFP-low: AFP <400 ng/ml, Model I: adjusted for propensity score, which was calculated by tumor diameter, tumor number, and extra-hepatic metastasis.

AFP, alpha‑fetoprotein; OS, overall survival; PFS, progress-free survival.

In AFP-high cohort, the median PFS was 3.9 (95% CI: 3.3–4.5) months, 7 (95% CI: 5.6–8.4) months, and 22.8 (95% CI: 13.9–NA) months for the high-stable, middle-stable, and sharp-falling groups, respectively (*P*<0.0001), as shown in Figure 3C. The median OS was 18.9 (95% CI: 5.9–31.9) months, 19 (95% CI: 13.9–24.1) months, and 22.8 (95% CI: 20.7–24.8) months for the high-stable, middle-stable, and sharp-falling groups, respectively (*P*<0.0001) (Fig. 3D). The multivariate HR of PFS and OS for AFP trajectories were presented in Table 4. Compared with the high-stable class, the middle-stable class had a lower risk of disease progression and death, but the lowest risk of disease progression and death was in the sharp-falling class in the unadjusted model. After adjusting tumor diameter, tumor number, and extra-hepatic metastasis, similar results were observed between AFP trajectory groups. In addition, the baseline characteristics after IPTW were shown in Supplementary Table S6 (Supplemental Digital Content 9, http://links.lww.com/JS9/C851).

**Table 4:** AFP trajectory groups and multivariate hazard ratios of clinical outcomes in AFP-high patients

	Event/*N*	Unadjusted	Model I
PFS			
High-stable	34/48	1	1
Middle-stable	64/147	0.31 (0.24–0.41)	0.3 (0.23–0.38)
Sharp-falling	5/65	0.04 (0.03–0.06)	0.04 (0.03–0.06)
OS			
High-stable	22/48	1	1
Middle-stable	45/147	0.62 (0.49–0.79)	0.55 (0.44–0.7)
Sharp-falling	6/65	0.16 (0.11–0.23)	0.16 (0.11–0.22)

AFP-high: AFP ≥400 ng/ml; Model I: adjusted for propensity score, which was calculated by tumor diameter, tumor number, and extra-hepatic metastasis.

AFP, alpha‑fetoprotein; OS, overall survival; PFS, progress-free survival.

The multivariable analysis in all patients before IPTW demonstrated that tumor number (*P*=0.002) and extra-hepatic metastasis (*P*<0.0001) were independent prognostic factors for PFS, and AFP level (*P*=0.029), tumor number (*P*=0.007) and extra-hepatic metastasis (*P*<0.0001) were independent predictive factors for OS. The results are shown in Supplementary Table S7 (Supplemental Digital Content 10, http://links.lww.com/JS9/C852).

**Figure 4:**
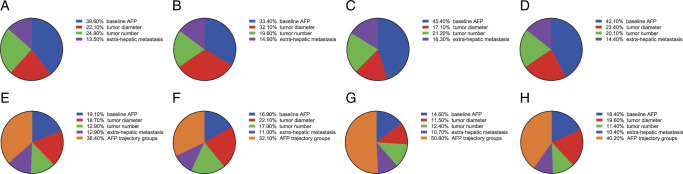
Relative importance of risk factors for clinical outcomes. The relative importance of risk factors for (A) PFS and (B) OS in AFP-low group. The relative importance of risk factors for (C) PFS and (D) OS in AFP-high group. The relative importance of risk factors plus AFP trajectory group for (E) PFS and (F) OS in AFP-low group. The relative importance of risk factors plus AFP trajectory group for (G) PFS and (H) OS in AFP-high group. AFP, alpha-fetoprotein; PFS, progression-free survival; OS, overall survival.

Moreover, we conducted an analysis to delineate the individual contributions of various parameters in predicting PFS and OS, including tumor diameter, tumor number, extra-hepatic metastasis, and baseline AFP levels (Fig. 4A–D). Upon incorporating the AFP trajectory classification, it became evident that its predictive power surpassed that of all conventional clinical parameters in relation to PFS and OS, rather in the AFP-low or AFP-high groups (Fig. 4E–H).

## Discussion

This longitudinal, real-world cohort study marks the pioneering endeavor in delineating the trajectories of AFP fluctuations and probing their correlation with clinical outcomes among HCC patients undergoing treatment with bevacizumab in combination with immunotherapy. Three distinct AFP trajectories persisted consistently throughout the observational period following the initial intervention. Moreover, we ascertained that AFP trajectories emerged as the paramount determinant in predicting both PFS and OS. The advantages of this present study are grounded in: (1) the incorporation of a genuine, broadened study cohort within China; (2) directing attention toward AFP dynamic changes as opposed to its singular value; (3) the delineation of AFP trajectories encompassing both AFP-low and AFP-high patient subgroups; and (4) the exhaustive documentation of immediate and enduring treatment outcomes among individuals afflicted with advanced HCC undergoing therapeutic intervention involving bevacizumab concomitant with immunotherapy.

A multitude of antecedent studies have elucidated the correlation between AFP levels and the prognosis for HCC patients, spanning from early-stage cases managed surgically to intermediate-stage instances treated with transarterial chemoembolization (TACE) and extending to advanced stages addressed through systemic therapeutic modalities^13–18,28–30^. The evidence of these retrospective studies suggested that AFP was the robust subclass across all stages of HCC. This study also found that AFP-low patients had better clinical outcomes than AFP-high patients. In addition, since the significant differences in the distributions as well as the clear variations of AFP among HCC individuals, we are concerned that HCC tumors may exhibit different biological characteristics and sensitivities to bevacizumab plus immunotherapy treatment among individuals with varying AFP levels. Therefore, in this study, we have divided patients into two groups, AFP-low and AFP-high, and performed model construction and following analysis separately. In AFP-low patients, three distinct trajectories have been delineated, designated as follows: high-rising (25%; *n*=69), low-stable (59.1%; *n*=163), and sharp-falling (15.9%; *n*=44). Similarly, in the AFP-high patients, three discernible trajectories have been identified, characterized as high-stable (18.5%; *n*=48), middle-stable (56.5%; *n*=147), and sharp-falling (25%; *n*=65). The discernible difference in trajectory patterns across patients with divergent AFP levels serves to underscore the imperative for stratification.

The relationship between changes in serum tumor biomarkers and prognosis has been recognized in many types of cancers. In colorectal cancer (CRC), Zhang *et al*. conducted a multicenter retrospective study that included 3539 CRC patients who underwent curative resection, and distinct trajectory groups were identified by the latent class growth mixed model. Patients were grouped into subgroups jointly by CEA, CA19-9, and CA125 according to preoperative levels and longitudinal trajectories, respectively. They found that dynamic measurements of CEA, CA19-9, and CA125 could monitor the survival of patients after surgery^23^. In HCC, the notion of AFP serological response delineates a distinct pathophysiological trajectory spanning all stages of HCC. The etiology of HCC, whether HBV, HCV, or alcohol-related, can influence AFP levels and treatment response. Understanding these differences is crucial for effective management and personalized treatment strategies for HCC patients. Among advanced HCC patients undergoing treatment with PD-(L)1 inhibitors in combination with antiangiogenic therapy, a reduction in AFP levels has emerged as a reliable biomarker heralding improved clinical outcomes. Specifically, thresholds of 20% for atezolizumab plus bevacizumab, 10% for nivolumab plus sorafenib, and 15% for nivolumab or pembrolizumab-TKIs (tyrosine kinase inhibitors) have been identified^31–33^. Despite variations in the definition of AFP response across studies, the collective evidence underscores its robustness as a predictor for clinical outcomes of HCC patients under immunotherapy. Consequently, we delineate the trajectory characterized by a sharp decline in AFP levels as the AFP serological response curve, positing it as a distinct pathophysiological process pertinent to immunotherapy. Of note, it was the sharp-falling cohort that exhibited the most favorable clinical outcomes. Despite the emergence of drug resistance and a subsequent rise in AFP levels, the outcomes of the sharp-falling cohort are better than other cohorts. This observation underscores the significance of the initial treatment response in determining patient prognosis. It further underscores the necessity of promptly reducing tumor burden through aggressive initial interventions. Additionally, it highlights the nonlinear relationship between AFP levels and disease severity or prognosis. Even when AFP levels are high prior to treatment initiation, their rapid reduction post-treatment can lead to favorable patient prognoses. For high-rising, middle-stable, high-stable, or high-rising cohorts, it becomes evident that the continuous administration of treatment does not yield significant reductions in AFP levels or may even result in an increment, thereby indicating the emergence of treatment resistance. Consequently, it becomes imperative to modify the treatment strategy at this juncture, thus preventing the squandering of the treatment time window and the escalation of economic burdens associated with persisting in drug administration despite its ineffectiveness. Collectively, this conceptualization of trajectory presents a novel and pragmatic approach, wherein clinicians may rely on the observation of AFP fluctuations in lieu of complex calculations, thereby facilitating clinical decision-making. Meanwhile, these results could provide a theoretical basis for the development of risk stratification tools incorporating AFP dynamics.

This study also possesses limitations. Firstly, this was a retrospective study conducted on a single-center cohort. Its retrospective design exposes it to potential selection bias. Although we enrolled relatively large numbers of patients and conducted IPTW to reduce the potential bias, a multicenter clinical trial is imperative to substantiate our findings. Secondly, the current delineation of three AFP trajectories was derived from data pertaining to the Chinese populace characterized by prevalent hepatitis B infection and cirrhosis. The nonproliferation class of HCC, more frequently associated with HCV infection and alcohol abuse, may exhibit distinct AFP trajectories. However, several studies have underscored AFP response as a predictor for clinical outcomes of HCC patients, which suggested that dynamic change of AFP could forecast the prognosis of systemic therapy in different classes of HCC patients. Finally, while the proposed method represents a novel and accessible approach for monitoring AFP dynamics in clinical settings, it is noteworthy that not all individuals align perfectly with the characteristics of the delineated AFP trajectory groups. Future multicenter studies with diverse HCC etiologies and larger sample sizes are needed to validate our findings and explore the prognostic value of AFP trajectories in combination with other biomarkers.

## Conclusion

Three discrete AFP trajectories in HCC patients undergoing bevacizumab and immunotherapy constituted an independent biomarker indicative of clinical outcomes. We reconfirm the prognostic relevance of AFP serological response as a biomarker conducive to enhancing the management of HCC patients. Rapid reduction of AFP after treatment is associated with a favorable prognosis regardless of subsequent changes in AFP, while sustained high levels of post-treatment AFP would lead to poor outcomes. Findings from this study hold potential clinical utility in dynamically forecasting the efficacy of immunotherapy combined with antiangiogenic therapy in HCC patients.

## Ethical approval

Following the ethical tenets outlined in the 1975 Declaration of Helsinki, this research endeavor was undertaken. The investigations involving human subjects received approval from the Institutional Review Board of our cancer center (Approval Number: B2023-673-01).

## Consent

Signed informed consent for data utilization was obtained from the patients.

## Source of funding

This work is funded by the China Postdoctoral Science Foundation (No: 2023M744018 to Y.Z. Fu), the Postdoctoral Fellowship Program of CPSF (No. GZC20233219 to Y.Z. Fu), the National Natural Science Foundation of China (No: 82372744 to Y.J. Zhang, 82103566 to D.D. Hu), Guangdong Basic and Applied Basic Research Foundation (2022A1515110961 to J.C. Wang), Guangzhou Science and Technology Plan Project (2023A04J2125 to J.C. Wang).

## Author contribution

All authors made a significant contribution to the work reported and approved the submitted manuscript. Z.Y.: conceptualization, data collection and curation, data analysis, investigation, methodology, and drafted and edited the manuscript; Y.F.: data collection and curation, methodology, and software; Q.W.: data collection and curation and methodology; Y.P., J.W., and J.C.: data collection and investigation; D.H. and Z.Z.: methodology and software; M.C. and Y.Z.: resources, conceptualization, supervision, review, and editing; Zhenyun Yang, Yizhen Fu and Qianyu Wang, should be considered joint first author.

## Conflicts of interest disclosure

The authors declare that they have no conflicts of interest in this work.

## Research registration unique identifying number (UIN)

1. Name of the registry: ResearchRegistry.com.
2. Unique identifying number or registration ID: researchregistry10187.
3. Hyperlink to your specific registration (must be publicly accessible and will be checked): not applicable.

## Guarantor

Yaojun Zhang.

## Data availability statement

The datasets used and/or analyzed during the current study are available from the corresponding author on reasonable request.

## Provenance and peer review

Not commissioned, externally peer-reviewed.

## Supplementary Material
