# Supplementary figures and images for: Dynamic changes of serum α-fetoprotein predict the prognosis of bevacizumab plus immunotherapy in hepatocellular carcinoma
Source: Int J Surg. 2024 Jun 21;111(1):751–60. doi: 10.1097/JS9.0000000000001860 (PMC11745582; doi:10.1097/JS9.0000000000001860)

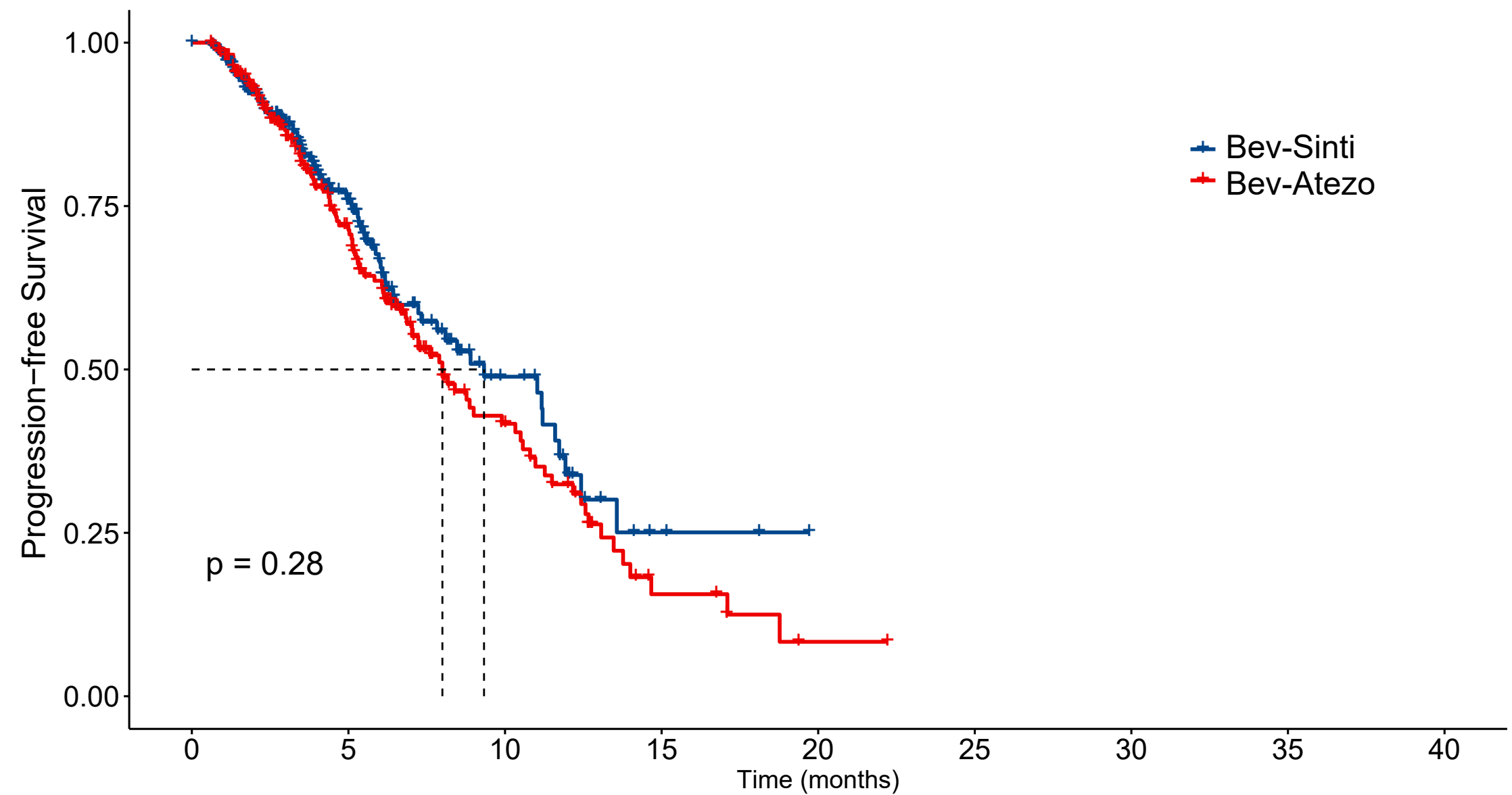

Number at risk

|           |     |     |    |   |   |   |   |   |
|-----------|-----|-----|----|---|---|---|---|---|
| Bev-Sinti | 290 | 96  | 22 | 3 | 0 | 0 | 0 | 0 |
| Bev-Atezo | 246 | 105 | 33 | 6 | 1 | 0 | 0 | 0 |

Time (months)

Supplement: Supplementary file 1 [file js9-111-0751-s001.pdf]

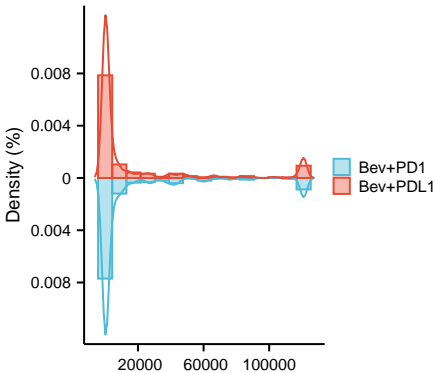

Supplement: Supplementary file 2 [file js9-111-0751-s002.pdf]

A

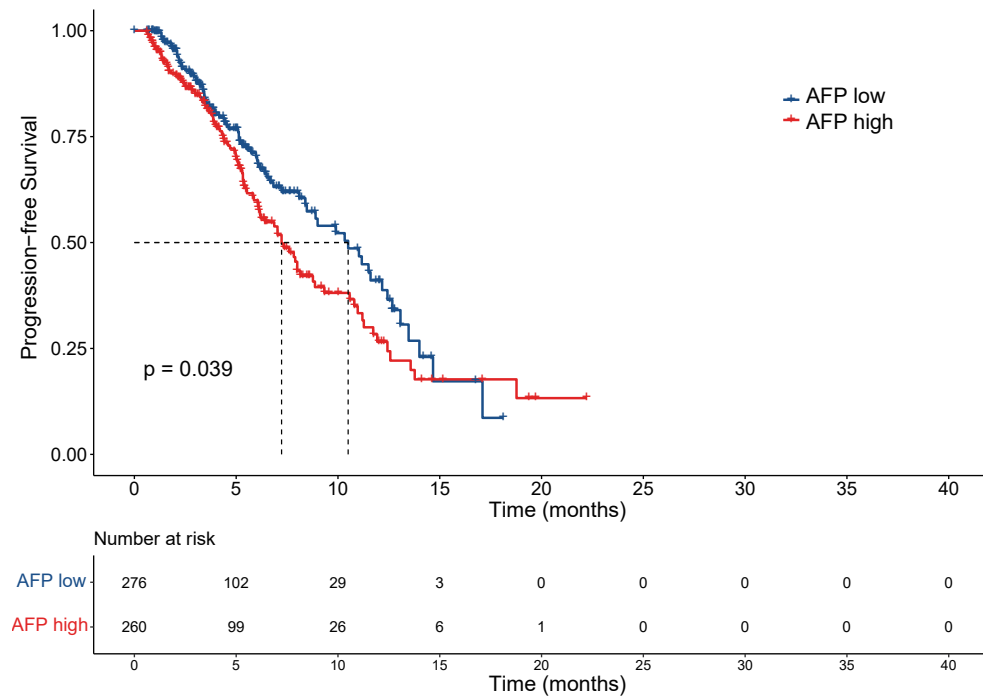

B

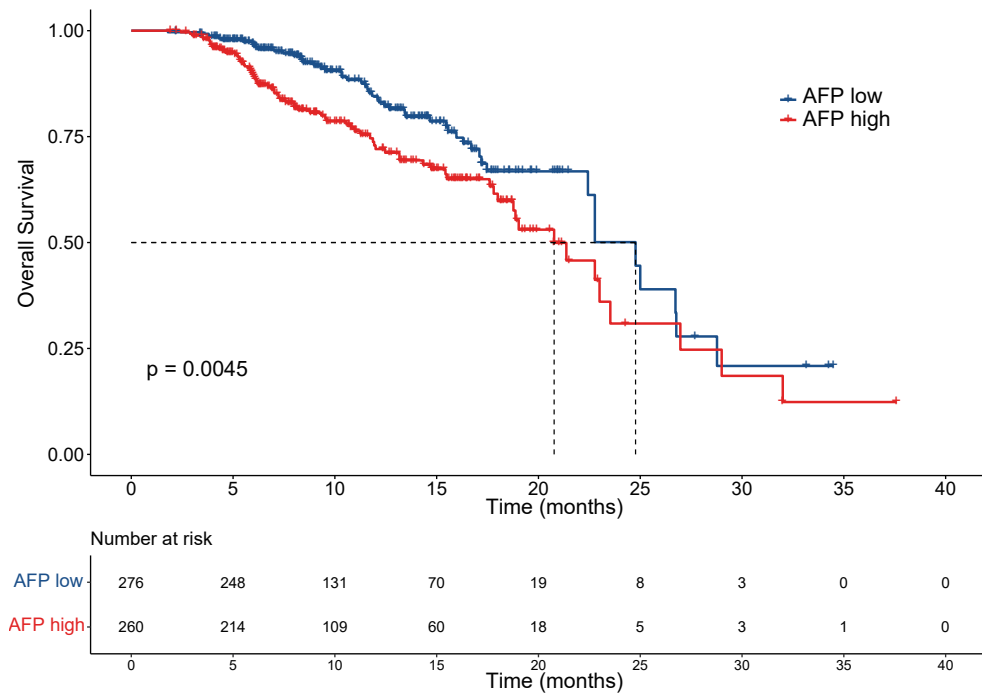

Supplement: Supplementary file 3 [file js9-111-0751-s003.pdf]
