# Supplementary material for: Dynamic changes of serum α-fetoprotein predict the prognosis of bevacizumab plus immunotherapy in hepatocellular carcinoma
Source: Int J Surg. 2024 Jun 21;111(1):751–60. doi: 10.1097/JS9.0000000000001860 (PMC11745582; doi:10.1097/JS9.0000000000001860)
Supplement: Supplementary file 4 [file js9-111-0751-s004.docx]

**Table S1: Baseline Characteristics of HCC Patients**

| **Variables** | **Bev-Sinti**  **(n=290)** | **Bev-Atezo**  **(n=246)** | ***P* value** |
| --- | --- | --- | --- |
| Age, years | 53.8 ± 11.4 | 53.3 ± 11.3 | 0.575 |
| Sex |  |  | 0.332 |
| Male | 259 (89.3) | 213 (86.6) |  |
| Female | 31 (9.7) | 33 (13.4) |  |
| Hepatitis infection |  |  | 0.753 |
| Yes | 252 (86.9) | 216 (87.8) |  |
| No | 38 (13.1) | 30 (12.2) |  |
| ALBI grade |  |  | 0.072 |
| I | 189 (65.2) | 173 (70.3) |  |
| II | 96 (33.1) | 73 (29.7) |  |
| III | 5 (1.7) | 0 (0) |  |
| Tumor diameter (cm) | 6.7 ± 4.3 | 6.7 ± 4.2 | 0.997 |
| Tumor number |  |  | 0.56 |
| Single | 76 (26.2) | 70 (28.5) |  |
| Multiple | 214 (73.8) | 176 (71.5) |  |
| Macrovascular invasion |  |  | 0.74 |
| Yes | 142 (49) | 124 (50.4) |  |
| No | 148 (51) | 122 (49.6) |  |
| Extra‑hepatic metastasis |  |  | 0.238 |
| Yes | 122 (42.1) | 116 (47.2) |  |
| No | 168 (57.9) | 130 (52.8) |  |
| ALT, IU/L | 36.9 (5.2-316.6) | 38.1 (3.3-258.5) | 0.484 |
| AST, IU/L | 51.5 (6.9-368.7) | 49.5 (15.2-432.7) | 0.457 |
| Albumin, g/L | 41.8 (21-51.7) | 42.4 (29-54.2) | 0.146 |
| TBil, μmol/L | 14.2 (4.7-349.1) | 15.1 (3.4-235.1) | 0.169 |
| WBC, ×10^9^/L | 6.7 (2-20.4) | 6.6 (2.2-17.1) | 0.411 |
| Hemoglobin, g/L | 140.5 (83-201) | 143 (75-185) | 0.301 |
| Platelet, ×10^9^/L | 201.5 (47-647) | 183 (52-643) | 0.184 |
| PT, seconds | 12 (9.9-18.3) | 12 (9.80-16) | 0.543 |

**Notes:** Data are presented as mean±SD, median (range), or n (%).

**Abbreviations:** Bev-Sinti, bevacizumab plus sintilimab; Bev-Atezo, bevacizumab plus atezolizumab; ALBI grade, Albumin-Bilirubin grade; ALT, alanine aminotransferase; AST, aspartate aminotransferase; TBil, total bilirubin; WBC, white blood cell; PT, prothrombin time.
