# Supplementary material for: Dynamic changes of serum α-fetoprotein predict the prognosis of bevacizumab plus immunotherapy in hepatocellular carcinoma
Source: Int J Surg. 2024 Jun 21;111(1):751–60. doi: 10.1097/JS9.0000000000001860 (PMC11745582; doi:10.1097/JS9.0000000000001860)
Supplement: Supplementary file 5 [file js9-111-0751-s005.docx]

**Table S2: Baseline Characteristics of HCC Patients**

| **Variables** | **AFP high**  **(n=260)** | **AFP low**  **(n=276)** | ***P* value** |
| --- | --- | --- | --- |
| Age, years | 51.8 ± 11.9 | 55.2 ± 10.5 | < 0.0001 |
| Sex |  |  | 0.799 |
| Male | 228 (87.7) | 244 (88.4) |  |
| Female | 32 (12.3) | 32 (11.6) |  |
| Hepatitis infection |  |  | 0.606 |
| Yes | 229 (88.1) | 239 (86.6) |  |
| No | 31 (11.9) | 37 (13.4) |  |
| ALBI grade |  |  | 0.614 |
| I | 171 (65.8) | 191 (69.2) |  |
| II | 87 (33.5) | 82 (29.7) |  |
| III | 2 (0.8) | 3 (1.1) |  |
| Tumor diameter (cm) | 7.3 ± 4.3 | 6.1 ± 4.1 | < 0.0001 |
| Tumor number |  |  | 0.002 |
| Single | 55 (21.2) | 91 (33) |  |
| Multiple | 205 (78.8) | 185 (67) |  |
| Macrovascular invasion |  |  | < 0.0001 |
| Yes | 152 (58.5) | 114 (41.3) |  |
| No | 108 (41.5) | 162 (58.7) |  |
| Extra‑hepatic metastasis |  |  | 0.537 |
| Yes | 119 (45.8) | 119 (43.1) |  |
| No | 141 (54.2) | 157 (56.9) |  |
| ALT, IU/L | 39.2 (3.3-268.2) | 36.5 (5.2-316.6) | 0.304 |
| AST, IU/L | 62.6 (15.2-373.5) | 44.4 (6.9-432.7) | < 0.0001 |
| Albumin, g/L | 41.7 (27.9-52.8) | 42.7 (21-54.2) | 0.237 |
| TBil, μmol/L | 15.6 (4.7-349.1) | 13.8 (3.4-235.1) | 0.003 |
| WBC, ×10^9^/L | 6.7 (2.4-20.4) | 6.6 (2-17.1) | 0.643 |
| Hemoglobin, g/L | 141 (76-201) | 141.5 (75-181) | 0.675 |
| Platelet, ×10^9^/L | 204 (52-643) | 184 (47-647) | 0.07 |
| PT, seconds | 12.1 (9.9-15.9) | 11.9 (9.8-18.3) | 0.074 |

Notes: Data are presented as mean±SD, median (range), or n (%).AFP high: AFP≥400 ng/ml; AFP low: AFP<400 ng/ml

**Abbreviations:** AFP alpha‑fetoprotein; ALBI grade, Albumin-Bilirubin grade; ALT, alanine aminotransferase; AST, aspartate aminotransferase; TBil, total bilirubin; WBC, white blood cell; PT, prothrombin time.
