# Supplementary material for: Dynamic changes of serum α-fetoprotein predict the prognosis of bevacizumab plus immunotherapy in hepatocellular carcinoma
Source: Int J Surg. 2024 Jun 21;111(1):751–60. doi: 10.1097/JS9.0000000000001860 (PMC11745582; doi:10.1097/JS9.0000000000001860)
Supplement: Supplementary file 6 [file js9-111-0751-s006.docx]

**Table S3: Parameters of the different latent class models during fitting process in the AFP-low group**

| **Model name** | **Number of trajectories** | **entropy** | **loglik** | **BIC** | **%class1** | **%class2** | **%class3** | **%class4** | **%class5** |
| --- | --- | --- | --- | --- | --- | --- | --- | --- | --- |
| lcga | 2 | 0.951504 | -1090.06 | 2225.08 | 25.36232 | 74.63768 | NA | NA | NA |
| ggm | 2 | 0.881915 | -814.988 | 1686.18 | 21.01449 | 78.98551 | NA | NA | NA |
| gm | 2 | 0.65714 | -658.405 | 1384.255 | 52.53623 | 47.46377 | NA | NA | NA |
| mlin | 2 | 0.689462 | -676.2 | 1414.223 | 76.81159 | 23.18841 | NA | NA | NA |
| mbeta | 2 | 0.748491 | -435.853 | 944.7703 | 76.44928 | 23.55072 | NA | NA | NA |
| mspl | 2 | 0.704461 | -382.472 | 854.8698 | 80.43478 | 19.56522 | NA | NA | NA |
| mspl5q | 2 | 0.700785 | -380.104 | 850.1342 | 80.07246 | 19.92754 | NA | NA | NA |
| lcga | 3 | 0.890342 | -937.762 | 1942.968 | 32.24638 | 22.82609 | 44.92754 | NA | NA |
| ggm | 3 | 0.852039 | -671.026 | 1426.357 | 17.3913 | 18.84058 | 63.76812 | NA | NA |
| gm | 3 | 0.715922 | -587.779 | 1271.104 | 45.65217 | 20.28986 | 34.05797 | NA | NA |
| mlin | 3 | 0.82154 | -621.819 | 1327.944 | 21.73913 | 21.37681 | 56.88406 | NA | NA |
| mbeta | 3 | 0.796161 | -368.74 | 833.027 | 25 | 15.94203 | 59.05797 | NA | NA |
| mspl | 3 | 0.638692 | -368.65 | 849.7089 | 38.76812 | 23.91304 | 37.31884 | NA | NA |
| mspl5q | 3 | 0.674682 | -362.591 | 837.5907 | 39.49275 | 23.91304 | 36.5942 | NA | NA |
| lcga | 4 | 0.890451 | -833.339 | 1756.604 | 13.04348 | 41.66667 | 32.24638 | 13.04348 | NA |
| ggm | 4 | 0.872619 | -620.616 | 1353.639 | 59.42029 | 17.75362 | 8.695652 | 14.13043 | NA |
| gm | 4 | 0.7305 | -524.982 | 1173.612 | 44.56522 | 27.17391 | 15.57971 | 12.68116 | NA |
| mlin | 4 | 0.624113 | -621.819 | 1350.425 | 21.73913 | 22.82609 | 55.43478 | 0 | NA |
| mbeta | 4 | 0.744689 | -341.84 | 801.7088 | 23.18841 | 14.49275 | 19.56522 | 42.75362 | NA |
| mspl | 4 | 0.674216 | -317.914 | 770.7173 | 18.84058 | 11.95652 | 39.13043 | 30.07246 | NA |
| mspl5q | 4 | 0.692203 | -311.664 | 758.2183 | 17.02899 | 12.68116 | 32.6087 | 37.68116 | NA |
| lcga | 5 | 0.843464 | -805.963 | 1724.334 | 14.85507 | 26.08696 | 9.057971 | 21.01449 | 28.98551 |
| ggm | 5 | 0.778407 | -579.77 | 1300.049 | 6.521739 | 52.89855 | 15.94203 | 15.57971 | 9.057971 |
| gm | 5 | 0.7049 | -510.695 | 1173.141 | 21.37681 | 13.04348 | 23.18841 | 27.89855 | 14.49275 |
| mlin | 5 | 0.805062 | -555.705 | 1240.679 | 7.971014 | 14.85507 | 11.23188 | 55.7971 | 10.14493 |
| mbeta | 5 | 0.05433 | -471.615 | 1083.741 | 23.18841 | 34.42029 | 3.985507 | 1.811594 | 36.5942 |
| mspl | 5 | 0.656344 | -314.788 | 786.9464 | 21.37681 | 12.31884 | 22.10145 | 27.89855 | 16.30435 |
| mspl5q | 5 | 0.730436 | -292.359 | 742.0892 | 0.724638 | 12.68116 | 16.30435 | 32.6087 | 37.68116 |

Abbreviations: lcga, model with fixed intercept and slope; ggm, model with random intercept; gm, model with random intercepts and random slopes; mlin, model with linear transformation; mbeta, model with rescaled β cumulative distribution; mspl, model with 5 equidistant nodes I-splines transformation; mspl5q, model with 5 quantiles nodes I-splines transformation; loglik, log-likelihood; BIC, Bayesian information criteria value; %class, percentage of patients in the corresponding class; NA, not applicable.

Among the above 7 parameters, lcga, ggm and gm belong to the latent class linear mixed model; mlin, mbeta, mspl and mspl5q belong to the latent process mixed model.

The model marked in red is the best model finally adopted.
