# Supplementary material for: Dynamic changes of serum α-fetoprotein predict the prognosis of bevacizumab plus immunotherapy in hepatocellular carcinoma
Source: Int J Surg. 2024 Jun 21;111(1):751–60. doi: 10.1097/JS9.0000000000001860 (PMC11745582; doi:10.1097/JS9.0000000000001860)
Supplement: Supplementary file 7 [file js9-111-0751-s007.docx]

**Table S4: Parameters of the different latent class models during fitting process in the AFP-high group**

| Model name | Number of trajectories | entropy | loglik | BIC | %class1 | %class2 | %class3 | %class4 | %class5 |
| --- | --- | --- | --- | --- | --- | --- | --- | --- | --- |
| lcga | 2 | 0.85391 | -1894.35 | 3833.184 | 56.53846 | 43.46154 | NA | NA | NA |
| ggm | 2 | 0.884937 | -1456.43 | 2968.467 | 76.15385 | 23.84615 | NA | NA | NA |
| gm | 2 | 0.908794 | -1293.09 | 2652.914 | 76.53846 | 23.46154 | NA | NA | NA |
| mlin | 2 | 0.91215 | -1293.37 | 2647.91 | 76.53846 | 23.46154 | NA | NA | NA |
| mbeta | 2 | 0.865728 | -1100.34 | 2272.978 | 73.84615 | 26.15385 | NA | NA | NA |
| mspl | 2 | 0.79474 | -1095.5 | 2279.977 | 71.15385 | 28.84615 | NA | NA | NA |
| mspl5q | 2 | 0.844002 | -1043.09 | 2175.148 | 74.23077 | 25.76923 | NA | NA | NA |
| lcga | 3 | 0.866084 | -1682.48 | 3431.684 | 25.38462 | 36.15385 | 38.46154 | NA | NA |
| ggm | 3 | 0.87025 | -1374.29 | 2831.991 | 70.38462 | 19.61538 | 10 | NA | NA |
| gm | 3 | 0.801842 | -1250.43 | 2595.389 | 16.15385 | 65.76923 | 18.07692 | NA | NA |
| mlin | 3 | 0.824076 | -1252.9 | 2589.216 | 66.53846 | 17.30769 | 16.15385 | NA | NA |
| mbeta | 3 | 0.756646 | -1057.7 | 2209.924 | 21.53846 | 61.92308 | 16.53846 | NA | NA |
| mspl | 3 | 0.697874 | -1042.3 | 2195.809 | 23.46154 | 60.38462 | 16.15385 | NA | NA |
| mspl5q | 3 | 0.793006 | -1025.65 | 2162.519 | 18.46154 | 56.53846 | 25 | NA | NA |
| lcga | 4 | 0.909396 | -1568.04 | 3225.06 | 6.153846 | 38.46154 | 18.84615 | 36.53846 | NA |
| ggm | 4 | 0.770585 | -1364.32 | 2839.863 | 12.30769 | 23.07692 | 62.69231 | 1.923077 | NA |
| gm | 4 | 0.836059 | -1231.39 | 2585.113 | 51.15385 | 21.15385 | 3.461538 | 24.23077 | NA |
| mlin | 4 | 0.810402 | -1234.19 | 2574.04 | 63.84615 | 3.076923 | 16.53846 | 16.53846 | NA |
| mbeta | 4 | 0.830074 | -1041.75 | 2200.273 | 21.53846 | 11.15385 | 44.61538 | 22.69231 | NA |
| mspl | 4 | 0.648723 | -1023.1 | 2179.647 | 21.15385 | 35 | 26.92308 | 16.92308 | NA |
| mspl5q | 4 | 0.654591 | -981.859 | 2097.174 | 20 | 39.23077 | 25 | 15.76923 | NA |
| lcga | 5 | 0.891347 | -1444.27 | 2999.75 | 18.07692 | 26.53846 | 23.84615 | 25.38462 | 6.153846 |
| ggm | 5 | 0.720539 | -1295.47 | 2729.96 | 19.61538 | 16.92308 | 50 | 10.38462 | 3.076923 |
| gm | 5 | 0.769449 | -1185.78 | 2521.692 | 2.692308 | 14.61538 | 38.07692 | 23.46154 | 21.15385 |
| mlin | 5 | 0.780913 | -1223.02 | 2573.945 | 1.153846 | 36.15385 | 8.846154 | 33.84615 | 20 |
| mbeta | 5 | 0.755438 | -1026.39 | 2191.796 | 20 | 40.76923 | 18.84615 | 2.692308 | 17.69231 |
| mspl | 5 | 0.700376 | -1010.35 | 2176.402 | 3.076923 | 20.76923 | 16.53846 | 39.61538 | 20 |
| mspl5q | 5 | 0.661619 | -969.787 | 2095.274 | 2.307692 | 53.07692 | 19.61538 | 12.69231 | 12.30769 |

Abbreviations: lcga, model with fixed intercept and slope; ggm, model with random intercept; gm, model with random intercepts and random slopes; mlin, model with linear transformation; mbeta, model with rescaled β cumulative distribution; mspl, model with 5 equidistant nodes I-splines transformation; mspl5q, model with 5 quantiles nodes I-splines transformation; loglik, log-likelihood; BIC, Bayesian information criteria value; %class, percentage of patients in the corresponding class; NA, not applicable.

Among the above 7 parameters, lcga, ggm and gm belong to the latent class linear mixed model; mlin, mbeta, mspl and mspl5q belong to the latent process mixed model.
