# Supplementary material for: Dynamic changes of serum α-fetoprotein predict the prognosis of bevacizumab plus immunotherapy in hepatocellular carcinoma
Source: Int J Surg. 2024 Jun 21;111(1):751–60. doi: 10.1097/JS9.0000000000001860 (PMC11745582; doi:10.1097/JS9.0000000000001860)
Supplement: Supplementary file 8 [file js9-111-0751-s008.docx]

**­­­Table S5: Baseline Characteristics of AFP-low Patients After IPTW**

| **Variables** | **high-rising**  **(n=274.6)** | **low-stable**  **(n=273.7)** | **sharp-falling**  **(n=277.4)** | **SMD** | ***P* value** |
| --- | --- | --- | --- | --- | --- |
| Tumor diameter (cm) | 8.1 ± 4.4 | 8.2 ± 4.6 | 8.1 ± 4.3 | 0.008 | 0.932 |
| Tumor number |  |  |  | 0.051 | 0.327 |
| Single | 52.7 (23.2) | 66 (24.1) | 70.2 (25.3) |  |  |
| Multiple | 210.9 (76.8) | 207.7 (75.9) | 207.2 (74.7) |  |  |
| Macrovascular invasion |  |  |  | 0.020 | 0.790 |
| Yes | 110.9 (40.4) | 108.1 (39.5) | 117.3 (42.3) |  |  |
| No | 163.7 (59.6) | 165.6 (60.5) | 160.1 (57.7) |  |  |
| Extra‑hepatic metastasis |  |  |  | 0.071 | 0.393 |
| Yes | 100.2 (36.5) | 85.1 (31.1) | 92.1 (33.2) |  |  |
| No | 174.4 (63.5) | 188.6 (68.9) | 185.3 (66.8) |  |  |

**Notes:** Data are presented as mean±SD or n (%).AFP low: AFP<400 ng/ml

**Abbreviations:** AFP alpha‑fetoprotein; IPTW, inverse probability of treatment weighting, SMD, standard mean difference.
