# Supplementary material for: Dynamic changes of serum α-fetoprotein predict the prognosis of bevacizumab plus immunotherapy in hepatocellular carcinoma
Source: Int J Surg. 2024 Jun 21;111(1):751–60. doi: 10.1097/JS9.0000000000001860 (PMC11745582; doi:10.1097/JS9.0000000000001860)
Supplement: Supplementary file 9 [file js9-111-0751-s009.docx]

**Table S6: Baseline Characteristics of AFP-high Patients After IPTW**

| **Variables** | **high-stable**  **(n=258.2)** | **middle-stable**  **(n=261.5)** | **sharp-falling**  **(n=259.3)** | **SMD** | ***P* value** |
| --- | --- | --- | --- | --- | --- |
| Tumor diameter (cm) | 9.1 ± 4.9 | 8.9 ± 4.7 | 9 ± 4.6 | 0.072 | 0.498 |
| Tumor number |  |  |  | 0.016 | 0.781 |
| Single | 52.4 (20.3) | 49.4 (18.9) | 55 (21.2) |  |  |
| Multiple | 205.8 (79.7) | 212.1 (81.1) | 204.3 (78.8) |  |  |
| Macrovascular invasion |  |  |  | 0.013 | 0.739 |
| Yes | 148.2 (57.4) | 152.2 (58.2) | 157.1 (60.6) |  |  |
| No | 110 (42.6) | 109.3 (41.8) | 102.2 (39.4) |  |  |
| Extra‑hepatic metastasis |  |  |  | 0.083 | 0.566 |
| Yes | 129.4 (50.1) | 129.7 (49.6) | 118.5 (45.7) |  |  |
| No | 128.8 (49.9) | 131.8 (50.4) | 140.8 (54.3) |  |  |

**Notes:** Data are presented as mean±SD or n (%).AFP high: AFP≥400 ng/ml

**Abbreviations:** AFP alpha‑fetoprotein; IPTW, inverse probability of treatment weighting, SMD, standard mean difference.
