# Supplementary material for: Dynamic changes of serum α-fetoprotein predict the prognosis of bevacizumab plus immunotherapy in hepatocellular carcinoma
Source: Int J Surg. 2024 Jun 21;111(1):751–60. doi: 10.1097/JS9.0000000000001860 (PMC11745582; doi:10.1097/JS9.0000000000001860)
Supplement: Supplementary file 10 [file js9-111-0751-s010.docx]

**Table S7: Univariate and Multivariate Cox Regression Analyses of Risk Factors for Progression Free Survival and Overall Survival in All Patients before IPTW**

| **Variables** | **PFS** | | | | **OS** | | | |
| --- | --- | --- | --- | --- | --- | --- | --- | --- |
|  | **Univariate** | | **Multivariate** | | **Univariate** | | **Multivariate** | |
|  | **HR(95% CI)** | ***P* value** | **HR(95% CI)** | ***P* value** | **HR(95% CI)** | ***P* value** | **HR(95% CI)** | ***P* value** |
| Age, y (>/≤50) | 0.89 (0.66-1.2） | 0.45 |  |  | 0.9 (0.63-1.29) | 0.57 |  |  |
| Gender(male/female) | 1.17 (0.76-1.82) | 0.48 |  |  | 0.91 (0.56-1.47) | 0.69 |  |  |
| Hepatitis(yes /no) | 1.31 (0.83-2.07) | 0.25 |  |  | 0.77 (0.48-1.24) | 0.28 |  |  |
| ALB, g/L, (>/≤35) | 0.54 (0.35-0.85) | 0.007 |  |  | 0.56 (0.32-0.98) | 0.043 |  |  |
| TBIL,umol/L,( >/≤17.1) | 1 (0.75-1.36) | 0.97 |  |  | 1.42 (1-2.03) | 0.052 |  |  |
| AFP,U/mL,(>/≤400) | 1.36 (1.01-1.82) | 0.04 |  |  | 1.61 (1.12-2.3) | 0.01 | 1.49 (1.04-2.15) | 0.029 |
| Largest tumor size (>/≤10 cm) | 1.06 (0.78-1.44) | 0.72 |  |  | 1.47 (1.03-2.11) | 0.036 |  |  |
| Tumor number (>1/1) | 1.88 (1.27-2.78) | 0.002 | 1.84 (1.24-2.73) | 0.002 | 2.33 (1.42-3.81) | 0.001 | 1.99 (1.21-3.28) | 0.007 |
| Macrovascular invasion (yes/no) | 1.07 (0.8-1.43) | 0.66 |  |  | 1.3 (0.91-1.85) | 0.15 |  |  |
| Extra-hepatic metastasis( yes/no) | 1.96 (1.46-2.64) | <0.0001 | 1.94 (1.44-2.61) | <0.0001 | 2.95 (2.02-4.3) | <0.0001 | 2.84 (1.94-4.14) | <0.0001 |

**Note:** P-value < 0.05 is statistically significant in both univariate and multivariate analyses

**Abbreviations:** ALB, albumin; TBIL, total bilirubin; AFP alpha‑fetoprotein.
